# (4*Z*)-4-[(2,6-Diisopropyl­anilino)(phen­yl)methyl­idene]-3-methyl-1-phenyl-1*H*-pyrazol-5(4*H*)-one

**DOI:** 10.1107/S1600536811000602

**Published:** 2011-01-12

**Authors:** Shu-Ling Lai, Ching-Yao Chang

**Affiliations:** a Department of Media and Design, Asia University, Taichung 413, Taiwan; b Department of Biotechnology, Asia University, Taichung 413, Taiwan

## Abstract

In the title compound, C_29_H_31_N_3_O, the three terminal benzene rings are oriented at dihedral angles of 20.7 (3), 65.8 (3) and 72.6 (3)° with respect to the central pyrazolone ring. Intra­molecular N—H⋯O hydrogen bonding occurs between the imine and carbonyl groups. Inter­molecular C—H⋯π inter­actions are present in the crystal structure.

## Related literature

For the catalysis of olefins polymerization by complexes containing *N,O*-bidentate ligands, see: Wang *et al.* (1998 ▶); Connor *et al.* (2003 ▶); Sun *et al.* (2003 ▶); Lü *et al.* (2006 ▶). For related structures, see: Wang *et al.* (2003 ▶); Li *et al.* (2009 ▶); Xu *et al.* (2010 ▶).
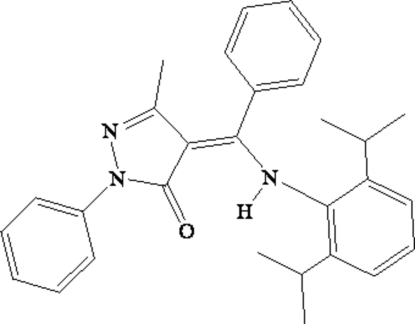


## Experimental

### 

#### Crystal data

C_29_H_31_N_3_O
*M* *_r_* = 437.57
Orthorhombic, 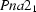

*a* = 12.9094 (6) Å
*b* = 10.6947 (5) Å
*c* = 17.9676 (8) Å
*V* = 2480.6 (2) Å^3^
*Z* = 4
Cu *K*α radiation
μ = 0.56 mm^−1^
*T* = 110 K
0.6 × 0.5 × 0.4 mm

#### Data collection

Oxford Diffraction Xcalibur Sapphire3 Gemini diffractometer
Absorption correction: multi-scan (*CrysAlis RED*; Oxford Diffraction, 2008 ▶) *T*_min_ = 0.702, *T*_max_ = 1.00
8437 measured reflections
3755 independent reflections
3643 reflections with *I* > 2σ(*I*)
*R*_int_ = 0.026

#### Refinement

*R*[*F* ^2^ > 2σ(*F* ^2^)] = 0.036
*wR*(*F* ^2^) = 0.100
*S* = 1.09
3755 reflections
302 parameters
1 restraint
H atoms treated by a mixture of independent and constrained refinement
Δρ_max_ = 0.27 e Å^−3^
Δρ_min_ = −0.21 e Å^−3^
Absolute structure: Flack (1983 ▶), 1293 Friedel pairs
Flack parameter: 0.2 (2)

### 

Data collection: *CrysAlis CCD* (Oxford Diffraction, 2005 ▶); cell refinement: *CrysAlis RED* (Oxford Diffraction, 2008 ▶); data reduction: *CrysAlis RED*; program(s) used to solve structure: *SHELXTL* (Sheldrick, 2008 ▶); program(s) used to refine structure: *SHELXTL*; molecular graphics: *SHELXTL*; software used to prepare material for publication: *SHELXTL*.

## Supplementary Material

Crystal structure: contains datablocks I, global. DOI: 10.1107/S1600536811000602/xu5120sup1.cif

Structure factors: contains datablocks I. DOI: 10.1107/S1600536811000602/xu5120Isup2.hkl

Additional supplementary materials:  crystallographic information; 3D view; checkCIF report

## Figures and Tables

**Table 1:** Hydrogen-bond geometry (Å, °) *Cg*1 and *Cg*2 are the centroids of the C18–C23 and N1,N2,C1–C3 rings, respectively.

*D*—H⋯*A*	*D*—H	H⋯*A*	*D*⋯*A*	*D*—H⋯*A*
N3—H3*A*⋯O	0.80 (2)	1.95 (2)	2.6734 (17)	149 (3)
C9—H9*A*⋯*Cg*1^i^	0.95	2.73	3.601 (2)	153
C14—H14*A*⋯*Cg*2^ii^	0.96	2.76	3.467 (2)	132

Symmetry codes: (i) 
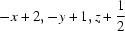
; (ii) 
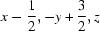
.

**Fig. 1.**
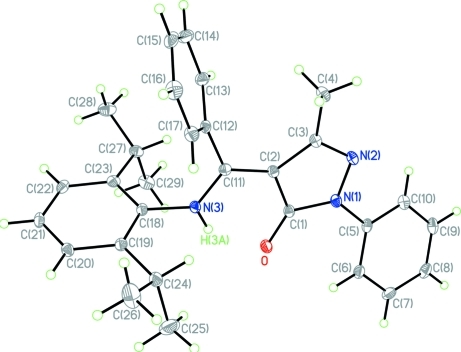
The molecular structure of (I), thermal ellipsoids for non-H atoms are shown at 25% probability levels.

## supplementary crystallographic information

### Comment

β-Ketoimine is an important family of *N,O*-bidentate ligands because of their ease of preparation and modification of steric and electronic effects. They are particularly interested for catalysis. For instance, their Cu complexes have shown to be effective in norbornene (NBE) polymerization (Lü *et al.*, 2006) in the presence of MAO and the Ni-based systems are used in ethylene polymerization (Wang *et al.*, 1998; Connor *et al.*, 2003; Sun *et al.*, 2003). As part of this work, the title compound (I) was prepared from β-diketone and 2,6-diisopropylaniline.

The molecular structure of (I) is shown in Fig. 1. The dihedral angles between the pyrazolone ring and C5–C10, C12–C17 and C18–C23 phenyl rings are 20.7 (3), 65.8 (3) and 72.6 (3)°, respectively. The C11–N3 distance of 1.333 (3) Å is similar to that for a C–N single bond (1.338 (8) Å (Li *et al.*, 2009) but is much longer than that for a C=N double bond (1.318 (3)Å) (Lü *et al.*, 2006) in pyrazolone compounds.

A strong intramolecular N(3)—H(3 A)···O hydrogen bond (Table 1.) is observed with N···O distance of 2.672 (2) Å, which is much shorter than their van der Waals radius. The intermolecular C—H···π interactions are present in the crystal structure (Table 1).

### Experimental

The title compound was synthesized by refluxing a mixture of 1-phenyl-3-methyl-4-benzoylpyrazol-5-one (10 mmol) and 2,6-diisopropylaniline (10 mmol) in ethanol (60 ml) for 16 h. Volatile materials were removed under vacuum and the residue was washed twice from cold ethanol solution to give yellow solids. The resulting solids were recrystallized from ethanol solution to yield yellow crystals after 2 d.

### Refinement

The imino H atom was located in a difference Fourier map and refined isotropically. Other H atoms were placed in idealized positions and constrained to ride on their parent atoms with C—H = 0.95–1.00 Å, *U_iso_*(H) = 1.5*U_eq_*(C) for methyl and 1.2*U_eq_*(C) for the others.

### Crystal data

**Table:**

C_29_H_31_N_3_O	*F*(000) = 936
*M**_r_* = 437.57	*D*_x_ = 1.172 Mg m^−^^3^
Orthorhombic, *P**n**a*2_1_	Cu *K*α radiation, λ = 1.54178 Å
Hall symbol: P 2c -2n	Cell parameters from 7431 reflections
*a* = 12.9094 (6) Å	θ = 4.1–71.6°
*b* = 10.6947 (5) Å	µ = 0.56 mm^−^^1^
*c* = 17.9676 (8) Å	*T* = 110 K
*V* = 2480.6 (2) Å^3^	Parallelpiped, yellow
*Z* = 4	0.6 × 0.5 × 0.4 mm

### Data collection

**Table:**

Oxford Diffraction Xcalibur Sapphire3 Gemini diffractometer	3755 independent reflections
Radiation source: Enhance (Cu) X-ray Source	3643 reflections with *I* > 2σ(*I*)
graphite	*R*_int_ = 0.026
Detector resolution: 16.0690 pixels mm^-1^	θ_max_ = 71.6°, θ_min_ = 4.8°
ω scans	*h* = −12→15
Absorption correction: multi-scan (*CrysAlis RED*; Oxford Diffraction, 2008)	*k* = −13→12
*T*_min_ = 0.702, *T*_max_ = 1.00	*l* = −22→18
8437 measured reflections	

### Refinement

**Table:**

Refinement on *F*^2^	Secondary atom site location: difference Fourier map
Least-squares matrix: full	Hydrogen site location: inferred from neighbouring sites
*R*[*F*^2^ > 2σ(*F*^2^)] = 0.036	H atoms treated by a mixture of independent and constrained refinement
*wR*(*F*^2^) = 0.100	*w* = 1/[σ^2^(*F*_o_^2^) + (0.080*P*)^2^] where *P* = (*F*_o_^2^ + 2*F*_c_^2^)/3
*S* = 1.09	(Δ/σ)_max_ < 0.001
3755 reflections	Δρ_max_ = 0.27 e Å^−^^3^
302 parameters	Δρ_min_ = −0.21 e Å^−^^3^
1 restraint	Absolute structure: Flack (1983), 1293 Friedel pairs
Primary atom site location: structure-invariant direct methods	Flack parameter: 0.2 (2)

### Special details

**Table:**

Geometry. All e.s.d.'s (except the e.s.d. in the dihedral angle between two l.s. planes) are estimated using the full covariance matrix. The cell e.s.d.'s are taken into account individually in the estimation of e.s.d.'s in distances, angles and torsion angles; correlations between e.s.d.'s in cell parameters are only used when they are defined by crystal symmetry. An approximate (isotropic) treatment of cell e.s.d.'s is used for estimating e.s.d.'s involving l.s. planes.
Refinement. Refinement of *F*^2^ against ALL reflections. The weighted *R*-factor *wR* and goodness of fit *S* are based on *F*^2^, conventional *R*-factors *R* are based on *F*, with *F* set to zero for negative *F*^2^. The threshold expression of *F*^2^ > σ(*F*^2^) is used only for calculating *R*-factors(gt) *etc*. and is not relevant to the choice of reflections for refinement. *R*-factors based on *F*^2^ are statistically about twice as large as those based on *F*, and *R*- factors based on ALL data will be even larger.

### Fractional atomic coordinates and isotropic or equivalent isotropic displacement parameters (Å^2^)

**Table:**

	*x*	*y*	*z*	*U*_iso_*/*U*_eq_	
O	0.91287 (8)	0.45238 (12)	0.24514 (7)	0.0298 (3)	
N1	0.93713 (10)	0.44539 (12)	0.37452 (8)	0.0264 (3)	
N2	0.87895 (11)	0.47065 (13)	0.43864 (8)	0.0277 (3)	
N3	0.72027 (10)	0.52807 (13)	0.21416 (8)	0.0249 (3)	
H3A	0.7776 (18)	0.502 (2)	0.2066 (14)	0.032 (5)*	
C1	0.88237 (12)	0.46862 (13)	0.31018 (10)	0.0252 (3)	
C2	0.78251 (11)	0.51329 (14)	0.33598 (10)	0.0243 (3)	
C3	0.78841 (12)	0.50848 (14)	0.41567 (10)	0.0252 (3)	
C4	0.70441 (13)	0.53150 (18)	0.47178 (11)	0.0333 (4)	
H4A	0.7326	0.5214	0.5221	0.050*	
H4B	0.6481	0.4714	0.4641	0.050*	
H4C	0.6777	0.6167	0.4658	0.050*	
C5	1.04093 (11)	0.40332 (14)	0.38254 (10)	0.0274 (3)	
C6	1.08976 (13)	0.34062 (15)	0.32476 (11)	0.0316 (4)	
H6A	1.0547	0.3269	0.2790	0.038*	
C7	1.19107 (14)	0.29789 (16)	0.33456 (12)	0.0368 (4)	
H7A	1.2252	0.2554	0.2951	0.044*	
C8	1.24239 (13)	0.31699 (16)	0.40159 (11)	0.0361 (4)	
H8A	1.3107	0.2861	0.4084	0.043*	
C9	1.19329 (14)	0.38119 (17)	0.45825 (12)	0.0365 (4)	
H9A	1.2288	0.3956	0.5038	0.044*	
C10	1.09248 (13)	0.42494 (17)	0.44944 (10)	0.0326 (4)	
H10A	1.0592	0.4691	0.4886	0.039*	
C11	0.70334 (12)	0.54389 (14)	0.28638 (9)	0.0227 (3)	
C12	0.59977 (12)	0.58874 (14)	0.31176 (9)	0.0250 (3)	
C13	0.58906 (14)	0.70320 (16)	0.34759 (11)	0.0330 (4)	
H13A	0.6477	0.7557	0.3542	0.040*	
C14	0.49253 (15)	0.74109 (18)	0.37387 (12)	0.0407 (4)	
H14A	0.4854	0.8194	0.3983	0.049*	
C15	0.40749 (14)	0.6652 (2)	0.36452 (13)	0.0447 (5)	
H15A	0.3418	0.6910	0.3827	0.054*	
C16	0.41744 (14)	0.5512 (2)	0.32857 (13)	0.0438 (5)	
H16A	0.3586	0.4989	0.3224	0.053*	
C17	0.51324 (13)	0.51316 (17)	0.30150 (11)	0.0322 (4)	
H17A	0.5196	0.4357	0.2761	0.039*	
C18	0.65579 (11)	0.56969 (14)	0.15362 (9)	0.0234 (3)	
C19	0.65238 (11)	0.69774 (14)	0.13647 (9)	0.0260 (3)	
C20	0.59414 (11)	0.73324 (15)	0.07474 (10)	0.0285 (3)	
H20A	0.5891	0.8193	0.0622	0.034*	
C21	0.54324 (11)	0.64521 (16)	0.03119 (10)	0.0296 (3)	
H21A	0.5043	0.6714	−0.0109	0.035*	
C22	0.54908 (12)	0.51948 (16)	0.04900 (10)	0.0285 (3)	
H22A	0.5144	0.4599	0.0187	0.034*	
C23	0.60517 (11)	0.47919 (15)	0.11070 (9)	0.0253 (3)	
C24	0.71736 (13)	0.79226 (16)	0.17900 (10)	0.0305 (4)	
H24A	0.7239	0.7617	0.2314	0.037*	
C25	0.82671 (14)	0.79704 (18)	0.14543 (12)	0.0387 (4)	
H25A	0.8560	0.7126	0.1442	0.058*	
H25B	0.8708	0.8512	0.1760	0.058*	
H25C	0.8231	0.8304	0.0947	0.058*	
C26	0.66815 (17)	0.92275 (18)	0.18176 (13)	0.0439 (5)	
H26A	0.5986	0.9168	0.2034	0.066*	
H26B	0.6633	0.9567	0.1312	0.066*	
H26C	0.7110	0.9781	0.2124	0.066*	
C27	0.61474 (14)	0.34042 (16)	0.12972 (11)	0.0331 (4)	
H27A	0.6263	0.3337	0.1846	0.040*	
C28	0.7098 (2)	0.2853 (2)	0.09084 (16)	0.0579 (6)	
H28A	0.7712	0.3345	0.1038	0.087*	
H28B	0.6994	0.2874	0.0368	0.087*	
H28C	0.7197	0.1986	0.1070	0.087*	
C29	0.5179 (2)	0.2645 (2)	0.11084 (16)	0.0581 (7)	
H29A	0.5289	0.1769	0.1247	0.087*	
H29B	0.5042	0.2701	0.0573	0.087*	
H29C	0.4586	0.2980	0.1384	0.087*	

### Atomic displacement parameters (Å^2^)

**Table:**

	*U*^11^	*U*^22^	*U*^33^	*U*^12^	*U*^13^	*U*^23^
O	0.0258 (5)	0.0416 (6)	0.0220 (6)	0.0060 (4)	−0.0012 (4)	0.0005 (5)
N1	0.0249 (6)	0.0309 (6)	0.0234 (7)	−0.0021 (5)	−0.0020 (5)	0.0028 (6)
N2	0.0303 (7)	0.0319 (7)	0.0208 (7)	−0.0063 (5)	−0.0024 (5)	0.0025 (6)
N3	0.0206 (6)	0.0307 (7)	0.0233 (7)	0.0047 (5)	−0.0019 (5)	−0.0008 (5)
C1	0.0231 (7)	0.0250 (7)	0.0274 (9)	−0.0023 (5)	−0.0018 (6)	0.0021 (6)
C2	0.0246 (7)	0.0242 (7)	0.0241 (9)	−0.0028 (5)	0.0005 (6)	0.0005 (6)
C3	0.0281 (7)	0.0252 (7)	0.0221 (8)	−0.0054 (6)	−0.0010 (6)	0.0009 (6)
C4	0.0324 (8)	0.0442 (10)	0.0232 (9)	−0.0026 (7)	0.0024 (7)	0.0020 (7)
C5	0.0267 (7)	0.0242 (6)	0.0314 (9)	−0.0038 (5)	−0.0055 (6)	0.0070 (6)
C6	0.0311 (8)	0.0282 (7)	0.0354 (10)	0.0029 (6)	−0.0109 (6)	−0.0003 (7)
C7	0.0336 (9)	0.0310 (8)	0.0459 (12)	0.0070 (7)	−0.0096 (8)	−0.0029 (8)
C8	0.0285 (8)	0.0316 (8)	0.0483 (12)	0.0022 (7)	−0.0113 (8)	0.0074 (7)
C9	0.0327 (8)	0.0411 (9)	0.0355 (10)	−0.0039 (7)	−0.0126 (7)	0.0075 (8)
C10	0.0298 (8)	0.0388 (9)	0.0292 (9)	−0.0037 (6)	−0.0039 (7)	0.0054 (7)
C11	0.0249 (7)	0.0207 (6)	0.0224 (8)	−0.0028 (5)	0.0003 (6)	−0.0002 (6)
C12	0.0266 (7)	0.0290 (7)	0.0194 (8)	0.0020 (5)	0.0012 (6)	0.0054 (6)
C13	0.0383 (9)	0.0283 (8)	0.0324 (9)	0.0034 (6)	0.0065 (7)	0.0028 (7)
C14	0.0475 (10)	0.0383 (8)	0.0363 (10)	0.0168 (7)	0.0092 (9)	0.0069 (9)
C15	0.0309 (9)	0.0629 (12)	0.0403 (11)	0.0185 (8)	0.0105 (7)	0.0127 (9)
C16	0.0273 (8)	0.0613 (12)	0.0428 (12)	−0.0018 (8)	0.0036 (7)	0.0079 (10)
C17	0.0279 (8)	0.0375 (8)	0.0310 (10)	−0.0024 (6)	0.0005 (7)	0.0020 (7)
C18	0.0198 (6)	0.0297 (7)	0.0205 (7)	0.0028 (5)	0.0020 (6)	0.0014 (6)
C19	0.0229 (7)	0.0300 (7)	0.0251 (8)	0.0009 (5)	0.0068 (6)	0.0015 (6)
C20	0.0272 (8)	0.0285 (7)	0.0297 (9)	0.0052 (6)	0.0074 (6)	0.0071 (7)
C21	0.0207 (7)	0.0421 (9)	0.0259 (8)	0.0055 (6)	−0.0001 (6)	0.0081 (7)
C22	0.0229 (7)	0.0365 (8)	0.0262 (9)	−0.0004 (6)	−0.0016 (6)	0.0002 (7)
C23	0.0224 (6)	0.0304 (8)	0.0231 (8)	0.0007 (5)	0.0014 (6)	0.0004 (6)
C24	0.0336 (8)	0.0295 (8)	0.0283 (9)	−0.0029 (6)	0.0052 (7)	−0.0005 (6)
C25	0.0325 (8)	0.0419 (9)	0.0418 (11)	−0.0094 (7)	0.0048 (8)	−0.0044 (8)
C26	0.0485 (11)	0.0328 (9)	0.0504 (13)	−0.0002 (7)	0.0082 (9)	−0.0057 (9)
C27	0.0433 (9)	0.0287 (8)	0.0272 (9)	−0.0015 (7)	−0.0068 (7)	0.0003 (7)
C28	0.0776 (16)	0.0373 (10)	0.0587 (16)	0.0200 (10)	0.0137 (12)	−0.0005 (10)
C29	0.0722 (15)	0.0434 (11)	0.0586 (15)	−0.0228 (10)	−0.0260 (12)	0.0142 (11)

### Geometric parameters (Å, °)

**Table:**

O—C1	1.245 (2)	C15—H15A	0.9500
N1—C1	1.378 (2)	C16—C17	1.390 (3)
N1—N2	1.402 (2)	C16—H16A	0.9500
N1—C5	1.421 (2)	C17—H17A	0.9500
N2—C3	1.304 (2)	C18—C19	1.404 (2)
N3—C11	1.327 (2)	C18—C23	1.400 (2)
N3—C18	1.440 (2)	C19—C20	1.393 (2)
N3—H3A	0.80 (2)	C19—C24	1.520 (2)
C1—C2	1.451 (2)	C20—C21	1.389 (3)
C2—C11	1.395 (2)	C20—H20A	0.9500
C2—C3	1.435 (2)	C21—C22	1.384 (2)
C3—C4	1.501 (2)	C21—H21A	0.9500
C4—H4A	0.9800	C22—C23	1.392 (2)
C4—H4B	0.9800	C22—H22A	0.9500
C4—H4C	0.9800	C23—C27	1.528 (2)
C5—C6	1.387 (3)	C24—C25	1.536 (2)
C5—C10	1.393 (2)	C24—C26	1.534 (2)
C6—C7	1.397 (2)	C24—H24A	1.0000
C6—H6A	0.9500	C25—H25A	0.9800
C7—C8	1.390 (3)	C25—H25B	0.9800
C7—H7A	0.9500	C25—H25C	0.9800
C8—C9	1.382 (3)	C26—H26A	0.9800
C8—H8A	0.9500	C26—H26B	0.9800
C9—C10	1.392 (3)	C26—H26C	0.9800
C9—H9A	0.9500	C27—C28	1.531 (3)
C10—H10A	0.9500	C27—C29	1.529 (3)
C11—C12	1.492 (2)	C27—H27A	1.0000
C12—C13	1.390 (2)	C28—H28A	0.9800
C12—C17	1.391 (2)	C28—H28B	0.9800
C13—C14	1.393 (2)	C28—H28C	0.9800
C13—H13A	0.9500	C29—H29A	0.9800
C14—C15	1.375 (3)	C29—H29B	0.9800
C14—H14A	0.9500	C29—H29C	0.9800
C15—C16	1.386 (3)		
			
C1—N1—N2	112.33 (12)	C12—C17—C16	119.86 (18)
C1—N1—C5	128.77 (15)	C12—C17—H17A	120.1
N2—N1—C5	118.88 (14)	C16—C17—H17A	120.1
C3—N2—N1	106.26 (13)	C19—C18—C23	122.60 (14)
C11—N3—C18	127.16 (14)	C19—C18—N3	119.03 (14)
C11—N3—H3A	111.2 (18)	C23—C18—N3	118.19 (14)
C18—N3—H3A	120.9 (19)	C20—C19—C18	117.23 (15)
O—C1—N1	126.87 (14)	C20—C19—C24	121.14 (15)
O—C1—C2	128.80 (15)	C18—C19—C24	121.40 (14)
N1—C1—C2	104.32 (14)	C21—C20—C19	121.27 (15)
C11—C2—C3	133.22 (15)	C21—C20—H20A	119.4
C11—C2—C1	121.61 (16)	C19—C20—H20A	119.4
C3—C2—C1	105.06 (14)	C22—C21—C20	120.15 (15)
N2—C3—C2	112.00 (15)	C22—C21—H21A	119.9
N2—C3—C4	119.07 (16)	C20—C21—H21A	119.9
C2—C3—C4	128.76 (15)	C21—C22—C23	120.86 (15)
C3—C4—H4A	109.5	C21—C22—H22A	119.6
C3—C4—H4B	109.5	C23—C22—H22A	119.6
H4A—C4—H4B	109.5	C22—C23—C18	117.87 (15)
C3—C4—H4C	109.5	C22—C23—C27	121.38 (15)
H4A—C4—H4C	109.5	C18—C23—C27	120.71 (14)
H4B—C4—H4C	109.5	C19—C24—C25	109.39 (14)
C6—C5—C10	120.58 (15)	C19—C24—C26	113.12 (15)
C6—C5—N1	120.37 (15)	C25—C24—C26	111.28 (15)
C10—C5—N1	119.05 (16)	C19—C24—H24A	107.6
C5—C6—C7	119.29 (17)	C25—C24—H24A	107.6
C5—C6—H6A	120.4	C26—C24—H24A	107.6
C7—C6—H6A	120.4	C24—C25—H25A	109.5
C8—C7—C6	120.51 (19)	C24—C25—H25B	109.5
C8—C7—H7A	119.7	H25A—C25—H25B	109.5
C6—C7—H7A	119.7	C24—C25—H25C	109.5
C9—C8—C7	119.53 (16)	H25A—C25—H25C	109.5
C9—C8—H8A	120.2	H25B—C25—H25C	109.5
C7—C8—H8A	120.2	C24—C26—H26A	109.5
C8—C9—C10	120.80 (17)	C24—C26—H26B	109.5
C8—C9—H9A	119.6	H26A—C26—H26B	109.5
C10—C9—H9A	119.6	C24—C26—H26C	109.5
C9—C10—C5	119.27 (18)	H26A—C26—H26C	109.5
C9—C10—H10A	120.4	H26B—C26—H26C	109.5
C5—C10—H10A	120.4	C28—C27—C23	109.70 (16)
N3—C11—C2	118.31 (14)	C28—C27—C29	110.5 (2)
N3—C11—C12	119.19 (14)	C23—C27—C29	113.57 (16)
C2—C11—C12	122.46 (15)	C28—C27—H27A	107.6
C13—C12—C17	119.56 (15)	C23—C27—H27A	107.6
C13—C12—C11	120.92 (14)	C29—C27—H27A	107.6
C17—C12—C11	119.49 (15)	C27—C28—H28A	109.5
C12—C13—C14	120.14 (17)	C27—C28—H28B	109.5
C12—C13—H13A	119.9	H28A—C28—H28B	109.5
C14—C13—H13A	119.9	C27—C28—H28C	109.5
C15—C14—C13	120.07 (19)	H28A—C28—H28C	109.5
C15—C14—H14A	120.0	H28B—C28—H28C	109.5
C13—C14—H14A	120.0	C27—C29—H29A	109.5
C14—C15—C16	120.14 (17)	C27—C29—H29B	109.5
C14—C15—H15A	119.9	H29A—C29—H29B	109.5
C16—C15—H15A	119.9	C27—C29—H29C	109.5
C15—C16—C17	120.22 (18)	H29A—C29—H29C	109.5
C15—C16—H16A	119.9	H29B—C29—H29C	109.5
C17—C16—H16A	119.9		
			
C1—N1—N2—C3	0.44 (16)	C2—C11—C12—C13	66.2 (2)
C5—N1—N2—C3	179.29 (13)	N3—C11—C12—C17	65.8 (2)
N2—N1—C1—O	−178.34 (15)	C2—C11—C12—C17	−111.77 (19)
C5—N1—C1—O	2.9 (2)	C17—C12—C13—C14	0.8 (3)
N2—N1—C1—C2	0.69 (15)	C11—C12—C13—C14	−177.13 (17)
C5—N1—C1—C2	−178.02 (13)	C12—C13—C14—C15	0.1 (3)
O—C1—C2—C11	0.8 (2)	C13—C14—C15—C16	−0.4 (3)
N1—C1—C2—C11	−178.21 (14)	C14—C15—C16—C17	−0.3 (3)
O—C1—C2—C3	177.56 (16)	C13—C12—C17—C16	−1.5 (3)
N1—C1—C2—C3	−1.45 (16)	C11—C12—C17—C16	176.53 (18)
N1—N2—C3—C2	−1.44 (17)	C15—C16—C17—C12	1.2 (3)
N1—N2—C3—C4	174.25 (14)	C11—N3—C18—C19	72.6 (2)
C11—C2—C3—N2	178.08 (16)	C11—N3—C18—C23	−112.15 (18)
C1—C2—C3—N2	1.86 (18)	C23—C18—C19—C20	1.2 (2)
C11—C2—C3—C4	2.9 (3)	N3—C18—C19—C20	176.16 (14)
C1—C2—C3—C4	−173.31 (15)	C23—C18—C19—C24	−173.31 (14)
C1—N1—C5—C6	−21.9 (2)	N3—C18—C19—C24	1.7 (2)
N2—N1—C5—C6	159.49 (14)	C18—C19—C20—C21	−1.2 (2)
C1—N1—C5—C10	158.71 (16)	C24—C19—C20—C21	173.28 (14)
N2—N1—C5—C10	−19.9 (2)	C19—C20—C21—C22	0.5 (2)
C10—C5—C6—C7	0.7 (3)	C20—C21—C22—C23	0.4 (2)
N1—C5—C6—C7	−178.67 (15)	C21—C22—C23—C18	−0.4 (2)
C5—C6—C7—C8	0.5 (3)	C21—C22—C23—C27	−178.27 (15)
C6—C7—C8—C9	−1.4 (3)	C19—C18—C23—C22	−0.4 (2)
C7—C8—C9—C10	1.2 (3)	N3—C18—C23—C22	−175.40 (14)
C8—C9—C10—C5	0.0 (3)	C19—C18—C23—C27	177.48 (15)
C6—C5—C10—C9	−1.0 (2)	N3—C18—C23—C27	2.5 (2)
N1—C5—C10—C9	178.42 (15)	C20—C19—C24—C25	−90.19 (18)
C18—N3—C11—C2	−170.51 (14)	C18—C19—C24—C25	84.08 (19)
C18—N3—C11—C12	11.8 (2)	C20—C19—C24—C26	34.5 (2)
C3—C2—C11—N3	−173.95 (17)	C18—C19—C24—C26	−151.26 (16)
C1—C2—C11—N3	1.8 (2)	C22—C23—C27—C28	89.8 (2)
C3—C2—C11—C12	3.7 (3)	C18—C23—C27—C28	−88.0 (2)
C1—C2—C11—C12	179.36 (13)	C22—C23—C27—C29	−34.4 (2)
N3—C11—C12—C13	−116.23 (18)	C18—C23—C27—C29	147.85 (19)

### Hydrogen-bond geometry (Å, °)

**Table:**

Cg1 and Cg2 are the centroids of the C18–C23 and N1,N2,C1–C3 rings, respectively.

**Table:**

*D*—H···*A*	*D*—H	H···*A*	*D*···*A*	*D*—H···*A*
N3—H3A···O	0.80 (2)	1.95 (2)	2.6734 (17)	149 (3)
C9—H9A···Cg1^i^	0.95	2.73	3.601 (2)	153
C14—H14A···Cg2^ii^	0.96	2.76	3.467 (2)	132

Symmetry codes: (i) −*x*+2, −*y*+1, *z*+1/2; (ii) *x*−1/2, −*y*+3/2, *z*.
